# Application of radiomics in adrenal incidentaloma: a literature review

**DOI:** 10.1007/s12672-022-00577-z

**Published:** 2022-10-28

**Authors:** Cheng Li, Yan Fu, Xiaoping Yi, Xiao Guan, Longfei Liu, Bihong T. Chen

**Affiliations:** 1 Department of Radiology, Xiangya Hospital, Central South University, Changsha, 410008 Hunan People’s Republic of China; 2 National Engineering Research Center of Personalized Diagnostic and Therapeutic Technology, Xiangya Hospital, Changsha, 410008 Hunan People’s Republic of China; 3 National Clinical Research Center for Geriatric Disorders (Xiangya Hospital), Central South University, Changsha , 410008 Hunan People’s Republic of China; 4 Hunan Key Laboratory of Skin Cancer and Psoriasis, Xiangya Hospital, Central South University, Changsha, 410008 Hunan People’s Republic of China; 5 Hunan Engineering Research Center of Skin Health and Disease, Xiangya Hospital, Central South University, Changsha, 410008 Hunan People’s Republic of China; 6 Department of Dermatology, Xiangya Hospital, Central South University, Changsha, 410008 Hunan People’s Republic of China; 7 Department of Urological Surgery, Xiangya Hospital, Central South University, Changsha, 410008 Hunan People’s Republic of China; 8 Department of Diagnostic Radiology, City of Hope National Medical Center, Duarte, CA USA

**Keywords:** Radiomics, Adrenal incidentaloma, Differentiation, Functional assessment, Texture analysis

## Abstract

Assessment of adrenal incidentaloma relies on imaging analysis and evaluation of adrenal function. Radiomics as a tool for quantitative image analysis is useful for evaluation of adrenal incidentaloma. In this review, we examined radiomic literature on adrenal incidentaloma including both adrenal functional assessment and structural differentiation of benign versus malignant adrenal tumors. In this review, we summarized the status of radiomic application on adrenal incidentaloma and suggested potential direction for future research.

**Supplementary Information:**

The online version contains supplementary material available at 10.1007/s12672-022-00577-z.

## Introduction

Adrenal incidentaloma (AI) is defined as an adrenal mass greater than 1 cm in diameter incidentally detected on imaging performed for indications other than evaluation for adrenal disease [1]. AI has been identified more frequently in recent years with a prevalence of 1.05–8.7%, which may be due to the increasing use of diagnostic imaging [2]. AI may have clinical consequences in two aspects, i.e., the biological behavior as benign versus malignant tumors, and the adrenal endocrine function. It is prudent to assess its secretory function and malignant nature, which affects treatment and prognosis of patients with AI [3].

Computed tomography (CT) is the most common imaging modality for diagnosis of AI and it is also the most useful non-invasive tool for assessing its malignant potential and endocrine function. Traditional imaging features favoring benign AI included the following: (1) size < 4 cm in diameter; (2) ≤ 10 Hounsfield units on CT; and (3) homogeneous density. However, these general features may only help with the initial evaluation of AI but not sufficient for endocrine function and malignant potential. For patients with AI, imaging evaluation and a subsequent endocrine testing have been recommended by most clinical guidelines [1, 4–7]. Developing a non-invasive strategy based on the initial CT could potentially assist decision-making and clinical management.

Radiomics was first introduced by Lambin et al. in 2012 [8] and refers to the high-throughput computational extraction of quantitative features from medical images. Radiomics generally consists of four steps: tumor segmentation, extraction and selection of features, model building, and model performance. It transforms the medical digital images into deep-level data for quantitative analysis [9–11]. Radiomics has been used for tumor classification, assessment of prognosis and recurrence, and evaluation of treatment effect in various cancers including cancers in lung, breast, thyroid and adrenal gland [12–14]. However, there is relative lack of literature on radiomics in AI. In this review, we assessed the current status of radiomic application for AI, and suggested future directions of radiomic research for AI.

## Adrenal incidentaloma (AI)

Most AIs are nonfunctioning benign tumors and their prevalence increases with age, reaching the highest in the 55–70 year age group [15]. Histologically, AIs are adrenal adenoma (41–52%), followed by metastatic diseases (19%), adrenocortical carcinoma (5–10%), myelolipoma (9%), and pheochromocytoma (8%) [16, 17]. There have been multiple guidelines for diagnosis and management of AI with complicated workflow including imaging methods to distinguish malignant from benign AI tumors and blood tests to assess adrenal endocrine function [1, 4–7]. The typical AI work-up has been time-consuming and costly (Figure S1).

## Literature search strategy

A literature search was conducted in PubMed including publications from year 2012 to year 2022. The search strategy included the following terms: ("logistic regression"[Title/Abstract] OR ("artificial intelligence"[Title/Abstract] OR "radiomics"[Title/Abstract] OR "deep learning"[Title/Abstract] OR "machine learning"[Title/Abstract])) AND (("incidentaloma"[Title/Abstract] OR "incidentalomas"[Title/Abstract]) AND "adrenal"[Title/Abstract]). The inclusion criteria were the following: (a) human studies on adrenal incidentaloma; (b) studies with imaging data; (c) studies with radiomic data and predictive modeling; and (d) studies published in any language. Exclusion criteria were the following: (a) secondary analyses only, without the primary imaging data; and (b) abstracts only, review articles, letters, case reports or editorials. No restrictions were placed on study design or population.

**Fig. 1:**
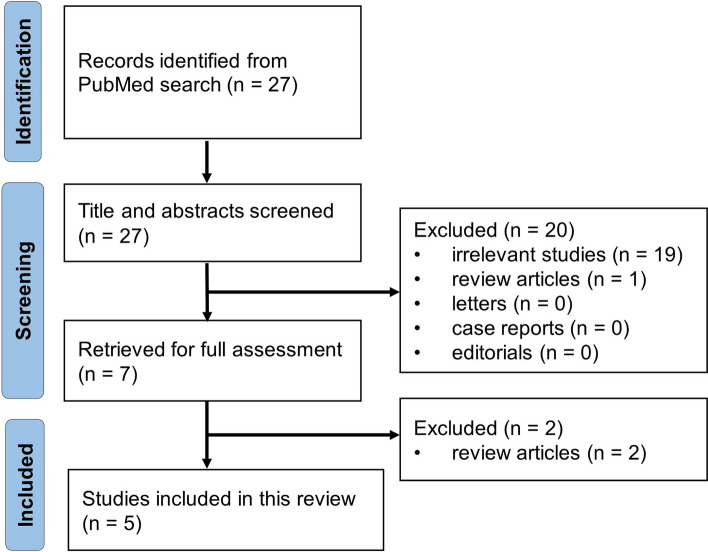
Study selection process

A total of 27 articles were initially identified through this strategy (Fig. 1). However, only 5 studies were deemed relevant after detailed review of the retrieved articles [18–21] and one of them was the latest research from our team [22]. After the initial literature search in PubMed, we identified 4 additional articles through manual search of the relevant literature [23–26]. Our manual search included searching other databases, retrieving the references cited in the included articles, and checking the radiomic literature, etc. The rationale for including these 4 additional articles not identified on the initial PubMed search was the following: these articles fulfilled the inclusion criteria as original imaging research on AI with detailed radiomics and modeling data from machine learning or deep learning methods.

## Radiomic method

Radiomics is a computational tool for medical imaging analysis and it may capture intra-tumoral heterogeneity and biological phenotypes [27]. Compare to clinical evaluation that relies on the physician’s personal expertise, the findings from radiomics through advanced statistical methods are more objective and repeatable.

**Fig. 2:**
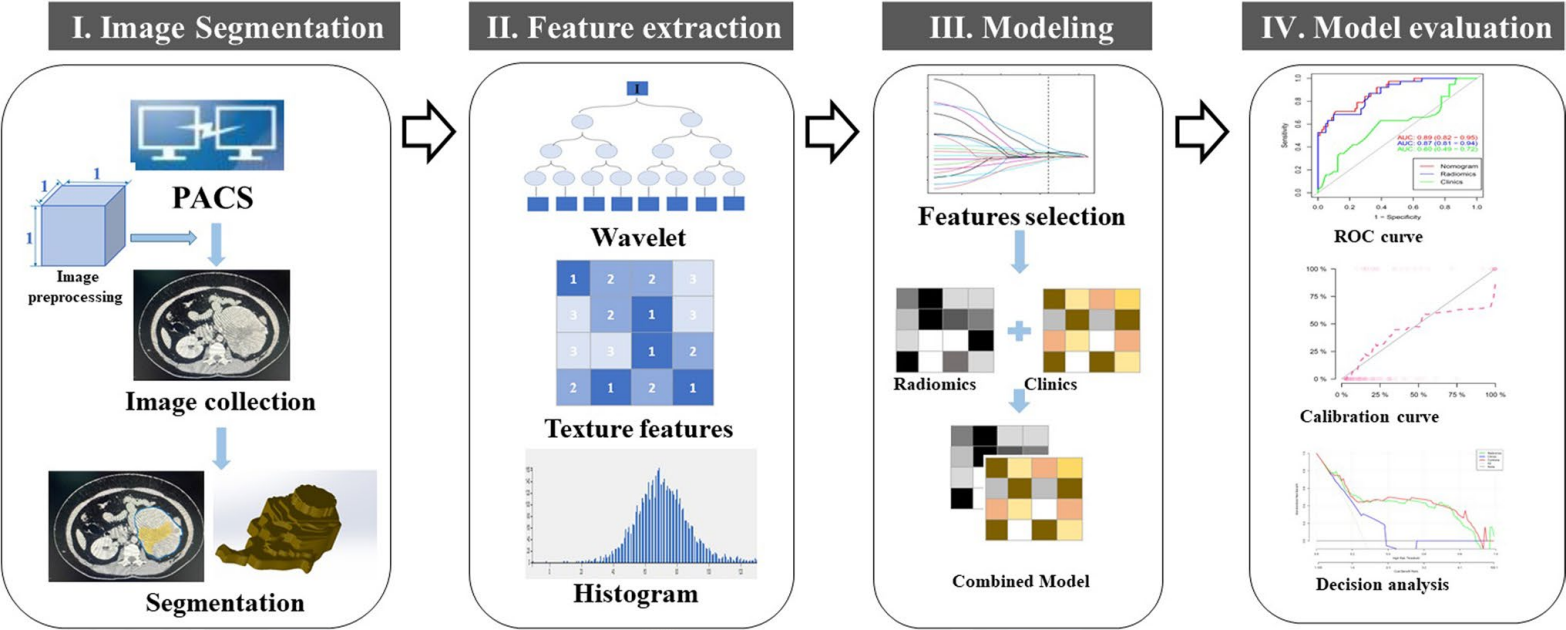
Flowchart showing workflow of radiomics for adrenal incidentaloma. I. Imaging segmentation. II. Features extraction. III. Modeling. IV. Model performance evaluation by receiver operating characteristic (ROC) curve, calibration curve, and decision curve analysis

Radiomic analysis generally consists of the following: (a) Imaging acquisition and volume of interest segmentation; (b) Extraction and selection of radiomic features; (c) Analysis and modeling; and (d) Evaluation of model performance, as presented in Fig. 2.

Accurate tumor segmentation through volume of interest (VOI) approach is critical for subsequent analysis. Manual, automatic, and semi-automatic segmentations are the three commonly used method for tumor segmentation [28, 29]. Manual segmentation by expert readers is often regarded as the “golden standard”. However, it is laborious and time-consuming, and there is also concern for variability between imagers [30]. Quantitative features can be extracted from the segmented tumors [9]. Second-order statistical features or textural features can quantify the intra-tumoral heterogeneity [31]. Textural features are computed from various matrices, but not calculated directly from the original image. The gray-level co-occurrence matrix (GLCM), the neighborhood gray-tone difference matrix (NGTDM), the gray level run-length matrix (GLRLM) and the Gray Level Size Zone Matrix (GLSZM) are typical parameters from textural feature analysis [32].

Following selection of radiomic features, a mathematical model can be built and used for predicting tumor type, treatment response, and prognosis. Support vector machines, decision trees, neural networks, linear and logistic regression are commonly used algorithms in radiomics [28, 33–35]. The classification efficiency of the model is generally evaluated with the receiver operating characteristic (ROC) curve, and the area under the curve (AUC). The consistency between the predicted classification results of the model and the actual classification is evaluated by calibration curve, and the clinical usefulness is evaluated by a decision curve analysis.

## Radiomic application on AI

A major challenge in clinical management of AI is to ascertain benign versus malignant nature of the tumor because the biological nature of AI affects its treatment and prognosis. The current research has mainly focused on the following two aspects of AI: endocrine functional assessment and differentiation of benign versus malignant tumors. An overview of the included studies is presented in Table 1.

**Table 1:** Summary of radiomic studies on adrenal incidentaloma

Study	Year	Radiologic technique	Classification/modeling method	Study design	No. of patients	No. of features		Result
						Initial	Final	
Yi [19]	2018	Unenhanced CT	Logistic multiple regression analysis	Retrospective study	108	377	30	Predictive model differentiating sPHEO from LPAs had an accuracy rate of 94.4% (sensitivity 86.2%; specificity 97.5%)
Yi [18]	2018	Enhanced CT Pre-enhanced CT	Logistic multiple regression analysis	Retrospective study	265	340	16	Differentiating sPHEO from LPAs with an AUC of 0.957/ 0.967(training/validation) based on one model, and 0.955/ 0.958(training/validation) based on another model
Liu [22]	2022	Enhanced CT Unenhanced CT	Logistic regression, support vector machine and random forest	Retrospective study	269	–	3	Best predictive model differentiating sPHEO from LPAs with an AUC of 0.919
Ho [23]	2019	Unenhanced CT Enhanced CT Chemical-shift MRI	Logistic multiple regression analysis	Retrospective study	20	–	21	Distinguishing LPAs from malignant adrenal nodules with a mean AUC of 0.8 (CECT texture features) and a mean AUC of 0.6 (CECT attenuation)
Elmohr [24]	2019	Unenhanced CT Enhanced CT	Random forest, Univariate logistic regression	Retrospective study	54	–	18	Differentiating between benign and malignant adrenal tumors with an AUC of 0.83 (Random forest) and an AUC of 0.89 (Univariate logistic regression)
Shi [25]	2019	Unenhanced CT Enhanced CT	ROC analysis	Retrospective study	225	–	6	Differentiating metastatic and benign adrenal masses with an AUC of 0.85
Yu [26]	2020	Enhanced CT	ROC analysis	Retrospective study	125	6	2	Differentiating benign from malignant adrenal tumors with a mean AUC of 0.96 based on entropy and a mean AUC of 0.93 based on standard deviation
Torresan [20]	2021	Unenhanced CT Enhanced CT	ROC analysis	Retrospective study	19	32	10	Differentiating adrenocortical adenoma from carcinoma
Moawad [21]	2021	Enhanced CT	Random forest	Retrospective study	40	3947	4	Differentiating between benign and malignant indeterminate adrenal lesions with an AUC of 0.85

*AUC* area under the receiver operating characteristic curve, *CT* computed tomography, *CECT* contrast enhanced CT, *LPAs* lipid-poor adenoma, *MRI* magnetic resonance imaging, *sPHEO* subclinical pheochromocytoma, *ROC* Receiver operating characteristic

### Endocrine function

Our own study by Yi et al. performed machine learning-based quantitative radiomic texture analysis on unenhanced CT to differentiate subclinical pheochromocytoma (sPHEO) from lipid-poor adenoma (LPAs) [19]. We selected 30 textural features on 80 LPAs from79 patients and 30 sPHEOs from 29 patients. Logistic multiple regression analysis was performed and our model had an accuracy rate of 94.4% with sensitivity 86.2% and specificity 97.5%. More importantly, this model was based on unenhanced CT which had the advantage over contrasted CT such as saving time, cost, avoiding additional radiation and potential complication from contrast enhancement.

In another study by our group, we distinguished sPHEO from LPAs based on both pre-enhanced and enhanced CT images [18]. Our study consisted of 265 consecutive patients [training cohort, 212 (LPA, 145; sPHEO, 67); validation cohort, 53 (LPA, 36; sPHEO, 17)]. Radiomic features were used to construct a radiomic signature (Rad-score) and a radiomic nomogram. The final model based on enhanced CT achieved an AUC of 0.957 in the training cohort and 0.967 in the validation cohort. The final model based on pre-enhanced CT resulted in an AUC of 0.955 in the training cohort and 0.958 in the validation cohort.

In a more recent publication from our group [22], we used CT-based machine learning models to differentiate sPHEO from LPAs, and our study included 188 tumors in the 183 patients with LPA and 92 tumors in 86 patients with sPHEO. Six imaging features were used to build models, and we assessed all combinations of these features and the three machine-learning models, i.e., logistic regression, support vector machine and random forest. Overall performance, conciseness and high sensitivity were the three parameters used to evaluate the model performance. Ultimately, the model consisting of features from pre-enhanced CT, tumor shape and necrosis/cystic degeneration with logistic regression approach performed the best, achieving an AUC of 0.919 and an accuracy of 0.859.

### Differentiation of benign versus malignant tumor

CT imaging features such as size, Hounsfield units and homogeneous density are the three common parameters for assessing the biological nature of AI tumors [15]. However, these general features would not work on special AI tumors. Taking LPA for example, most LPAs have CT attenuation values over 10 HU, similar to other adrenal tumors. A study by Ho et al. focused on distinguishing the LPAs from malignant adrenal nodules and they found that chemical-shift MRI could be useful for this purpose [23]. Specifically, they assessed 23 adrenal nodules in 20 patients, and obtained 21 second-order texture features. Except the unenhanced CT attenuation of the AI tumors, other imaging features revealed notable differences between benign and malignant adrenal nodules. Their model showed that contrast-enhanced CT and chemical-shift MRI were predictive of malignant AI (p = 0.003 and p = 0.02, respectively). This was the first study using chemical-shift MRI for distinguishing the LPAs from malignant adrenal masses. However, the study was limited by a small sample size.

A study by Elmohr [24] used quantitative CT texture analysis to differentiate between large adrenal adenomas and carcinomas in a cohort of 54 patients. Interestingly, they compared the accuracy of the prediction model to that of the radiologists. Their study revealed that combination of CT texture analysis and CT attenuation values, was likely to improve imaging evaluation by radiologists. A study by Shi [25] included 225 patients with 265 adrenal tumors (101 metastases, 98 pheochromocytomas, and 66 lipid-poor adenomas) for differentiating metastases from benign adrenal masses. Their study showed significant differences between metastases and benign adrenal masses in the texture parameters including the standard deviation (SD), entropy, mean of positive pixels and kurtosis on unenhanced images, and mean, SD of pixel distribution histogram, mean of positive pixels, and entropy on enhanced images. Their best performing model yielded a mean AUC of 0.85 with four texture parameters mentioned above on enhanced images. A study by Yu [26] performed texture analysis for differentiation between benign from malignant adrenal lesions on enhanced CT for a cohort of 125 patients. Their study showed that entropy and standard deviation were the two imagine features with significant differences between benign and malignant tumors. Entropy demonstrated a mean AUC of 0.96 and standard deviation demonstrated a mean AUC of 0.925 for discriminating tumors. These studies have a common limitation in that malignant adrenal lesions consisted of mostly metastases and were hence heterogeneous from various primary cancers.

A study by Torresan et al. used CT radiomics to differentiate between adrenocortical adenoma and carcinoma on 19 patients (9 adenomas, 10 carcinomas) [20]. First-order features and second-order features were extracted by principal component analysis [36]. In addition, they applied a K-means clustering technique, an unsupervised machine learning approach in their data analysis and accurately predicted malignancy in 7 of 8 adrenocortical carcinomas. In another study by Moawad [21], they used enhanced CT and machine learning algorithm to determine the pathology of the adrenal tumors in 40 patients. Their binary classification model using a random forest algorithm showed an AUC = 0.85 (sensitivity 84.2%, and specificity 71.4%).

The findings from our review were generally in agreement with the more recent literature and a review on the topic by Crimì et al. who performed a systematic review on CT radiomic texture analysis in AI [37]. Their review included 9 papers with a pooled median AUC of 0.85, indicating a diagnostic accuracy of 93% in differentiating adrenal adenoma from adrenocortical carcinomas. Our current review also included 9 papers but mostly different papers yet our results were similar to theirs with our AUC ranging from 0.8 to 0.96 for differentiating benign from malignant adrenal tumors such as adrenal metastases. In addition, our review was complimentary to their study as we also provided an assessment of the endocrine functioning adrenal tumors in addition to structural differentiation between benign and malignant AI tumors. For instance, we assessed studies including research from our own group on radiomic CT texture analysis for differentiating pheochromocytoma from atypical adrenal adenoma with low lipid [19]. Our own machine learning-based radiomic studies showed high performance of our prediction models for classifying pheochromocytoma from lipid-poor adenoma with AUC reaching 0.95. Overall, our studies and other’s data all suggest that CT radiomics may be used as a non-invasive tool for assessing both endocrine function and imaging characterization of AI.

The guideline set by European Society of Endocrinology for management of adrenocortical carcinoma specifically indicates the importance of imaging for diagnosis and treatment of adrenocortical carcinoma [38]. A CT scan of chest abdomen and pelvis is recommended for all patients with high suspicion for adrenocortical carcinoma. In addition, preoperative imaging and postoperative follow-up imaging are also recommended for patients undergoing treatment for adrenocortical carcinoma. Radiomics is a computational analysis of the existing imaging data and it generates multiple higher-order features for detailed characterization of adrenocortical carcinoma. Radiomics does not acquire additional imaging beyond what has already obtained per recommendations by the guidelines. Since the existing imaging complies with the guideline for management of adrenocortical carcinoma, the subsequent radiomic analysis of this imaging data should also meet the criteria of the guidelines for management of adrenocortical carcinoma.

In summary, all studies included in this review applied radiomics on CT including both unenhanced and enhanced CT images [18–26]. This could be due to CT being the most commonly used modality for initial imaging of abdominal pathology. In this review, one study included only unenhanced CT in their radiomic analysis [19], and two studies included only enhanced CT [21, 26], while the remaining studies reported radiomics on both unenhanced and enhanced CT images from texture analysis. All studies except one [20], utilized ROC and AUC to assess the accuracy of their radiomic results. The highest AUC was 0.96 by Yu et al. [26], while the lowest AUC was 0.8 by Ho et al. [23], and the mean AUC was 0.88 among all included studies in this review. Taken together, the radiomic analysis based on CT imaging of AI was robust and could be potentially useful in clinical management of AI. Nevertheless, more work needs to be done to improve its efficacy for broader clinical application.

## Limitation

There were two main limitations of this review. First, the studies reviewed here had one common issue. i.e., lack of interpretability. For instance, results from radiomic analysis may not be directly correlated with underlying tumor pathology and treatment response. Second, the studies in this review may have subjected to selection bias because of its retrospective nature. In addition, small sample size was a common problem among the included studies and therefore the generalizability of the radiomic findings was limited.

## Future direction

Functional assessment and differentiation of benign versus malignant tumors are the two main points for diagnosis and management of AI. The main challenge of managing functional AI tumors pertains to appropriate diagnosis and treatment of autonomous cortisol secretion [2]. Predicting the risk of distant metastasis and recurrence, genotyping of AI tumors such as pheochromocytoma and monitoring treatment response are important aspects for personalized patient care. Fortunately, most of the radiomic approaches have been studied extensively in various cancers [12, 14], which should be adoptable for AI tumors. For example, Huynh et al. analyzed CT images of 113 patients with stage I–II non-small cell lung cancer treated with stereotactic body radiation therapy. Their study showed that radiomic features had the potential to be prognostic for outcomes that conventional imaging metrics could not do in patients with radiation treatment [39]. Hence, similar radiomic approach may be applied to identify prognostic factors in AI tumors. Another potential future direction is in the management of AI tumors. Arshad et al. conducted a multi-center study and found that PET-CT based radiomics could provide potential information for risk classification of non-small cell lung cancer patients with radiotherapy and chemo-radiotherapy [40]. Future radiomic studies on management of autonomous cortisol secretion, risk factors and prognostic prediction of AI tumors are needed to advance the field and to improve clinical decision making.

## Conclusion

This review supports radiomics being a potentially useful non-invasive imaging tool for differentiating benign from malignant adrenal tumors. More studies need to be done to overcome the common issues with all radiomic applications such as the lack of explainability related to the tumor heterogeneity and to improve personalized care for patients with AI.

## Supplementary Information

Additional file1 (JPG 255 KB) Figure S1. Flowchart summary of existing guidelines in diagnosing and management of patients with adrenal incidentalomas. The main differences among the guidelines are in imaging and hormone testing.

## Data Availability

All data including codes will be available to qualified researchers.
