# Supplementary figures and images for: Application of radiomics in adrenal incidentaloma: a literature review
Source: Discov Oncol. 2022 Oct 28;13:112. doi: 10.1007/s12672-022-00577-z (PMC9616972; doi:10.1007/s12672-022-00577-z)

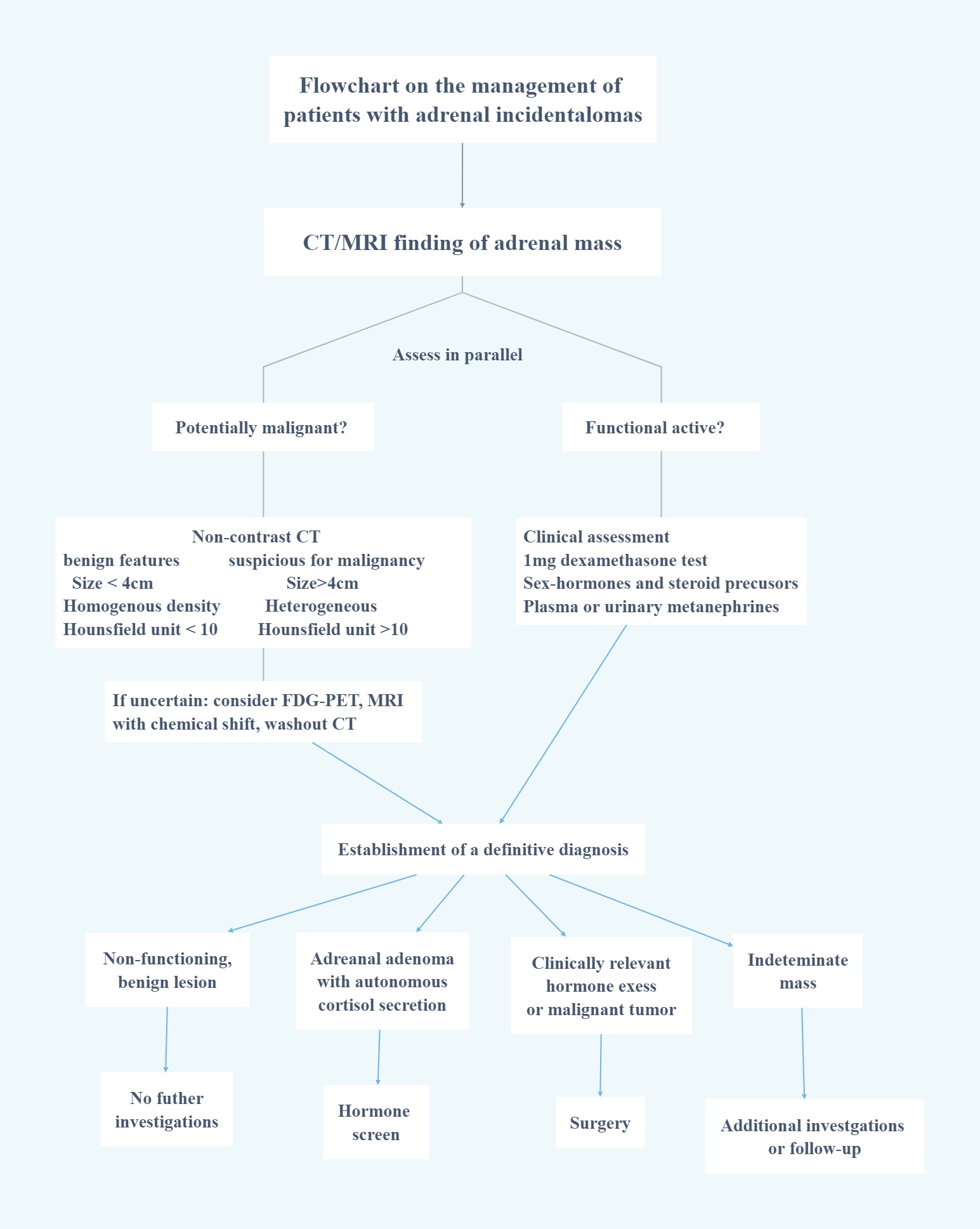

Supplement: Supplementary file 1 — Additional file1 (JPG 255 KB) Figure S1. Flowchart summary of existing guidelines in diagnosing and management of patients with adrenal incidentalomas. The main differences among the guidelines are in imaging and hormone testing. [file 12672_2022_577_MOESM1_ESM.jpg]
